# Experimental FTIR-MI and Theoretical Studies of Isocyanic Acid Aggregates

**DOI:** 10.3390/molecules28031430

**Published:** 2023-02-02

**Authors:** Justyna Krupa, Maria Wierzejewska, Jan Lundell

**Affiliations:** 1 Faculty of Chemistry, University of Wrocław, F. Joliot-Curie 14, 50-383 Wrocław, Poland; 2 Department of Chemistry, University of Jyväskylä, FI-40014 Jyväskylä, Finland

**Keywords:** HNCO, hydrogen bond, Fourier transform infrared (FTIR), matrix isolation (MI), solid argon, vibrational spectroscopy, intermolecular interaction, computational chemistry, molecular complex, atmospheric chemistry

## Abstract

Homoaggregates of isocyanic acid (HNCO) were studied using FTIR spectroscopy combined with a low-temperature matrix isolation technique and quantum chemical calculations. Computationally, the structures of the HNCO dimers and trimers were optimized at the MP2, B3LYPD3 and B2PLYPD3 levels of theory employing the 6-311++G(3df,3pd) basis set. Topological analysis of the electron density (AIM) was used to identify the type of non-covalent interactions in the studied aggregates. Five stable minima were located on the potential energy surface for (HNCO)_2_, and nine were located on the potential energy surface for (HNCO)_3_. The most stable dimer (D1) involves a weak, almost linear N-H⋯N hydrogen bond. Other structures are bound by a N-H⋯O hydrogen bond or by O⋯C or N⋯N van der Waals interactions. Similar types of interactions as in (HNCO)_2_ were found in the case of HNCO trimers. Among nine stable (HNCO)_3_ structures, five represent cyclic forms. The most stable T1 trimer structure is characterized by a six-membered ring formed by three N-H⋯N hydrogen bonds and representing high symmetry (C_3h_). The analysis of the HNCO/Ar spectra after deposition indicates that the N-H⋯O hydrogen-bonded dimers are especially prevalent. Upon annealing, HNCO trimers were observed as well. Identification of the experimentally observed species relied on previous experimental data on HNCO complexes as well as computed data on HNCO homoaggregates’ vibrational spectra.

## 1. Introduction

Isocyanic acid (HNCO) is an atmospheric pollutant emitted into the air during different processes such as fossil fuel combustion and biomass burning [1,2,3,4,5]. As a trace gas in ambient air, HNCO poses a health risk for humans since, when inhaled, the molecule participates in protein carbamylation reactions that lead to development of serious diseases [6]. In addition to research related to the chemistry of the atmosphere and the impact of HNCO on human health, properties and photochemical reactivity of isocyanic acid and its isomers have been extensively studied both theoretically and experimentally [7,8,9,10,11,12,13,14,15,16,17,18,19,20,21,22,23,24,25]. Four open-chain isomers were previously identified for CHNO species: fulminic acid (HCNO), isofulminic acid (HONC), cyanic acid (HOCN) and the most stable form, isocyanic acid (HNCO). Teles et al. [14] reported infrared spectra of all these four CHNO isomers together with their isotopomers isolated in low-temperature argon matrices. Theoretically, it has been shown by Shapley and Bacskay [23] that in addition to chain forms, several cyclic and branched CHNO structures of much higher energies are stable. Photolysis of HNCO has also been the subject of extensive studies both in argon and xenon matrices leading to isomerization and photodecomposition processes [8,17,18,26,27].

Although the properties of HNCO and its isomers are relatively well understood, much less data is available on molecular complexes of isocyanic acid. Such studies are useful to obtain information on how spectral properties and chemical reactivity of the molecule change upon intermolecular interactions. Raunier et al. [28] investigated the thermal reactivity of HNCO with water ice and the 1:1 HNCO complex with H_2_O isolated in an argon matrix using infrared spectroscopy. It was concluded, based on both experimental findings and the results of MP2/6-31G(d,p) calculations, that HNCO acts as a proton donor in the complex with H_2_O to form a N-H⋯O hydrogen bond. In turn, photo-induced decomposition of formohydroxamic acid (HCONHOH) isolated in solid argon led to the formation of two different forms of the 1:1 HNCO complex with water in which HNCO acted either as a proton donor or proton acceptor [29]. Molecular complexes of HNCO with NH_2_OH or CH_3_OH have been identified upon photodecomposition of N-hydroxyurea and acetohydroxamic acid, respectively [30,31]. Keresztes et al. [32] performed photolysis using 220 nm light on 1,2,5- and 1,3,4-oxadiazoles, and HCN⋯HNCO and HCN⋯HOCN complexes were produced upon photolysis. Subsequent photoreactions using excitation with a hydrogen lamp led to the formation of three complexes of isocyanic acid: HCNO⋯HNC, HNCO⋯HNC and HNC⋯HOCN. Two papers on HNCO complexes with dinitrogen and sulfur dioxide isolated in argon matrices have been published [33,34]. In both cases, 1:1 complexes exhibited either N-H⋯N or N-H⋯O hydrogen bonds. Along with them, complexes bound only by van der Waals interactions were detected. Very recently, Zhao et al. [35] reported computational studies on various hydrogen-bonded HNCO complexes, including the HNCO dimer, related to atmospheric chemistry. 

Non-covalent interactions are considered to have a significant influence on properties of chemical and biological systems. They also have an impact on atmospheric chemistry, affecting existing reactions and contributing to new reaction channels. Molecular complexes formed in dense environments at low temperature can affect chemistry of interstellar media and some planetary atmospheres. Here we report results of isocyanic acid aggregation in low-temperature argon matrices. The formation of dimers and trimers was experimentally followed by FTIR spectroscopy, and it was thereafter identified with the help of quantum chemical calculations. To our knowledge, such aggregates have not been the subject of experimental studies and could be of interest for better understanding of chemical reactivity of tropospheric gases, especially via intermolecular interactions of N-H-containing molecules. In our studies on HNCO aggregates, an interesting question arose as to which atoms in the acid are the best proton acceptor positions for hydrogen bonds involving the N-H group in the HNCO molecule. 

## 2. Results and Discussion

### 2.1. Structure and Energetics of HNCO Dimers

**Figure 1:**
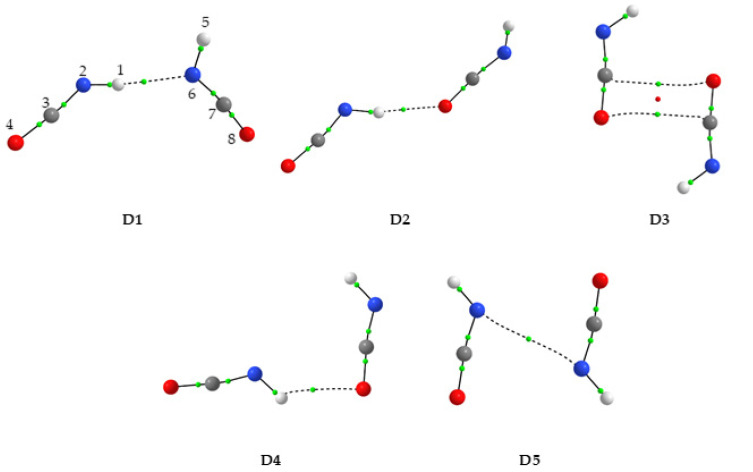
The MP2-optimized structures of the HNCO dimers. The H, N, C and O atoms are shown in white, blue, gray and red, respectively. The positions of the bond (3,−1) and ring (3,+1) critical points derived from AIM calculations are shown by small green and red dots, respectively.

Five minima (D1-D5) were found on the potential energy surface for the HNCO dimer at the MP2 and B3LYPD3 levels of theory. At the B3LYP and B2PLYPD3 levels, one of the structures denoted D4 converged to D2. All MP2-computed energy minima structures are shown in Figure 1 together with the atom numbering used in this work. Table 1 shows the values of two topological AIM parameters: the electron density ρ(r) and its Laplacian ∇^2^ρ(r) at the bond critical points. Table 1 also contains the MP2 calculated values of intermolecular distances and angles. Table 2 presents energetic parameters obtained for HNCO dimers using the MP2, B3LYP, B3LYPD3 and B2PLYPD3 methods. Based on the computed structures shown in Figure 1 and the values of the AIM parameters in Table 1, it was found that three structures (D1, D2 and D4) comprised hydrogen bonds of the N-H⋯N or N-H⋯O type, and all three possessed C_1_ symmetry. Two other structures (D3 and D5) were connected by van der Waals interactions and had C_2h_ symmetry. The most stable structure with the largest interaction energy (E_int_) appeared to be an open dimer D1 with two subunits bound by an almost linear N-H⋯N hydrogen bond, with the N⋯N distance of ca. 3.1 Å and the N-H⋯N angle of ca. 174°.

The two molecular moieties in D1 did not lie in the same plane but were twisted with respect to each other. The MP2 method gave a C3N2N6C7 dihedral angle of 62.0°, whereas B3LYPD3 and B2PLYPD3 predicted the D1 structure to be deviated from planarity by 78.3° and 70.7°, respectively. The D2 and D3 dimers were of almost equal stability compared with each other. The D2 form was an elongated structure in which the N-H group of one molecular moiety interacts with the terminal oxygen atom of the second subunit. The N-H⋯O hydrogen bond formed was close to linearity with the N-H⋯O angle of ca. 170°. The NCO backbones of the two moieties in D2 were twisted by ca. 25° from one another. A cyclic planar D3 structure with C_2h_ symmetry had, according to the AIM results, HNCO subunits connected by two C⋯O van der Waals interactions, and their backbones were anti-parallel to each other. The D4 dimer was also planar and contained, like D2, the N-H⋯O type hydrogen bond. However, in this case, the hydrogen bond appeared strongly bent with the N-H⋯O angle of ca. 131° and the backbones of the two moieties almost perpendicular to each other (the N2C7N6 angle of ca. 115°). From the energy point of view, the least stable D5 dimer of C_2h_ symmetry was characterized by anti-parallel NCO backbones, and hydrogen atoms were oriented away from the center of symmetry of the dimer. The two HNCO moieties were bound by one identified N⋯N van der Waals interaction.

Based on computational data on energetics for the HNCO dimers presented in Table 2, some observations can be made. At all levels of calculations employed, the D1 dimer was the most stable one and was characterized by the highest interaction energy. The three dimers D2, D3 and D4 were of similar energetic stability with respect to each other, and they were only slightly less stable than the global minimum D1. The D5 form was the least energetically favored, as this structure was bound only by van der Waals interactions. Very recently, four HNCO dimers of similar structures optimized at B3LYPD3/aug-cc-pVTZ level were reported [35]. These structures resemble the planar structures D1, D2 and D3 in this study. The two structures D4 and D5 were identified for the first time in this work and were not considered in the previous study [35]. 

It is interesting to compare the geometry of (HNCO)_2_ with the results obtained earlier for dimers of the sulfur analogue of isocyanic acid, HNCS [36]. Computational studies revealed three structures to be stable for (HNCS)_2_ (see Figure S1 in Supplementary Materials). Two of them exhibited N-H⋯N and N-H⋯S hydrogen bonds corresponding to the binding patterns found for D1 and D4 isocyanic acid dimers. Although the mutual arrangement of the two molecular moieties in these dimers is slightly different, the type of interaction is analogous. The other (HNCO)_2_ structures considered here do not have their counterparts in the case of isothiocyanic acid. There was a cyclic structure similar to structure D3 here with anti-parallel HNCS subunits, but the interaction was through N-H⋯S hydrogen bonds opposite to the C⋯O van der Waals interactions appearing in the HNCO dimer. Moreover, for the HNCS dimers [36] the contribution of dispersion to stabilize the system is crucial, and computational methods taking it into account are the right choice, while the results obtained at the more traditional B3LYP level are less reliable. To check whether this is also the case for HNCO dimers, i.e., if the dispersion effects have a significant contribution to the total energy of the interaction, we performed calculations using the B3LYP functional with the 6-311++G(3df,3pd) basis set. These data, shown in Table 2, indicated the highest contribution of dispersion one should expect for the cyclic D3 and D5 dimers characterized by two O⋯C or one N⋯N van der Waals interactions. The numbers in parentheses in Table 2 indicate the percentage contribution of dispersion to the total interaction energy.

### 2.2. Structure and Energetics of HNCO Trimers

**Figure 2:**
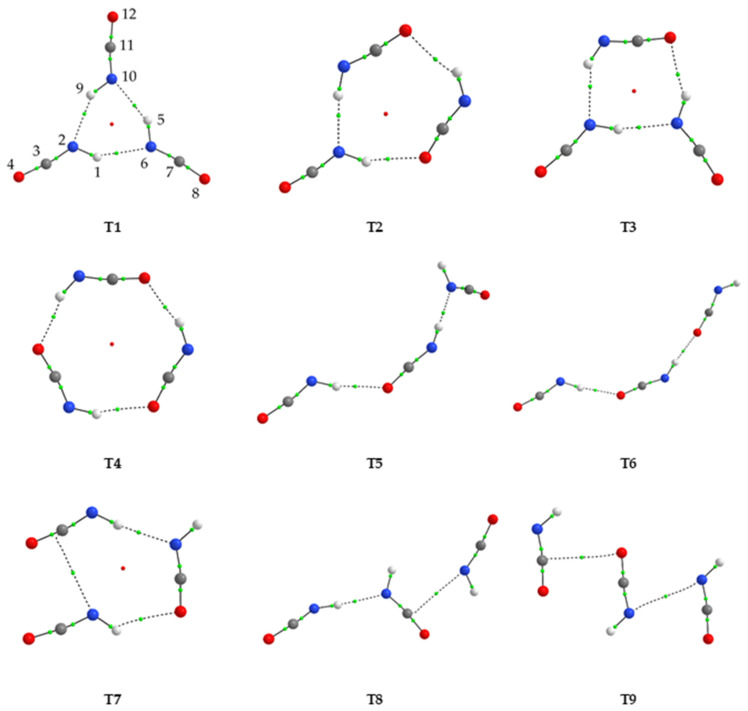
The MP2-optimized structures of the HNCO trimers. The H, N, C and O atoms are shown in white, blue, gray and red, respectively. The positions of the bond (3,−1) and ring (3,+1) critical points derived from AIM calculations are shown by small green and red dots, respectively.

Nine structures were found to be stable at all three levels of theory for HNCO trimers. Their geometries are shown in Figure 2, and their energetic parameters are presented in Table 3. The values of the topological AIM parameters and values of intermolecular distances and angles calculated at the MP2/6-311++G(3df,3pd) level for the trimers are gathered in Table S1 in the Supplementary Materials.

The geometry of (HNCO)_3_ is, as in the case of dimers, determined by the presence of hydrogen bonds and/or van der Waals interactions. Of the nine structures, the most stable form was a cyclic trimer T1 characterized by three N-H⋯N hydrogen bonds and C_3h_ symmetry. Trimers T2, T3 and T4 exhibited cyclic structures with a C_s_, C_s_ and C_3_ symmetry, respectively. They had very similar relative energies and were slightly less stable than T1. These trimers included N-H⋯O or both N-H⋯N and N-H⋯O hydrogen bonds. Another cyclic structure T7 characterized by C_1_ symmetry was the least stable of all hydrogen-bonded forms.

There were also four trimers that represent open, elongated structures with three moieties connected by hydrogen bonds (T5 and T6, both with C_1_ symmetry) or van der Waals contacts (T9, C_s_) or both (T8, C_1_). Optimized trimer structures indicated the existence of energetic co-operativity in the species. This was also illustrated by the calculated interaction energies. For example, for the most stable trimer (T1), the total interaction energy was about three times the interaction energy associated with a single hydrogen bond found for the D1 dimer. Other trimers with lower symmetry (T2 and T3), representing cyclic structures and containing both N-H⋯N and N-H⋯O hydrogen bonds, had similar energies as T1 and T4 structures. When the cyclic structures were open, the cooperativity effect mediated by the linked hydrogen bonds was lost, and the trimer structures appeared less stable and more weakly bound.

### 2.3. Results of Matrix Isolation FTIR Studies

**Figure 3:**
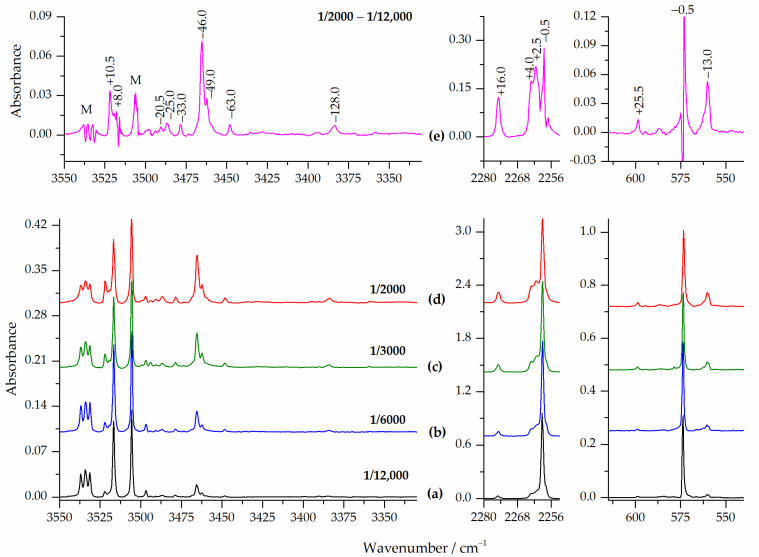
Bottom panels: the νNH, ν_as_NCO and δNCO regions in the spectra of HNCO/Ar matrices with 1/12,000 (**a**), 1/6000 (**b**), 1/3000 (**c**) and 1/2000 (**d**) concentrations. Top panels: the difference spectrum obtained by subtracting the HNCO/Ar = 1/12,000 spectrum from the HNCO/Ar = 1/2000 spectrum (**e**). The numbers show the red (minus) and blue (plus) wavenumber shifts compared to the HNCO monomer values. Letter M denotes HNCO monomer bands.

Figure 3 presents the selected regions of the infrared spectra of the HNCO/Ar matrices obtained upon deposition at 15 K (10 K for measurement) at different sample concentrations. Trace (e) in this figure shows the corresponding spectral ranges of the difference spectrum obtained by subtracting the HNCO/Ar = 1/12,000 spectrum from the HNCO/Ar = 1/2000 spectrum. When the HNCO/Ar mixtures were deposited on a cold window, the HNCO monomer bands appeared in the spectra, as previously described [12,14]. Note, that in the νNH stretching region of HNCO, a doublet is present due to the Fermi resonance [14]. In addition, even at high dilutions of the gaseous mixtures, several other absorptions were observed. The intensity of these bands increased relative to the monomer absorptions as the concentration of isocyanic acid in the matrix increased. 

**Figure 4:**
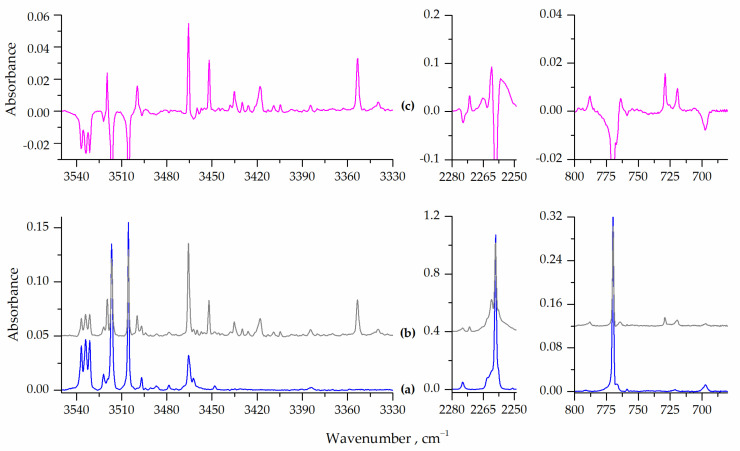
Bottom panels: the νNH, ν_as_NCO and δNCO vibrational spectral regions for HNCO/Ar = 1/6000 after deposition (**a**, blue line) and after subsequent 10 min annealing at 33 K (**b**, grey line). Top panels: the difference spectrum (**c**, pink line) obtained by subtracting the spectrum (**a**) from the spectrum (**b**).

Analyzing the spectra after the subsequent annealing processes of the matrices at 26, 28, 30, 33 and 35 K (the highest temperature possible for argon matrices annealing) revealed interesting changes in the bands observed. The temperature increase in the range of 26–30 K did not cause significant changes in the spectra, apart from a slight general increase in the observed intensities of the bands. More pronounced changes were observed upon annealing the matrix at 33 and 35 K. Figure 4 shows selected regions of the spectra of the HNCO/Ar = 1/6000 matrices obtained after deposition at 15 K and subsequent 10 min annealing at 33 K. The trace (c) in Figure 4 shows the corresponding ranges of the difference spectrum obtained by subtracting the spectrum after deposition from the spectrum after annealing at 33 K.

As can be seen in Figure 4 (trace (b)) there are bands which were not present or were relatively weak in the spectrum recorded after deposition (see trace (a)). Moreover, these additional bands grew in intensity when the matrix was annealed up to 33 K. Therefore, they must be related to dimers or higher aggregates of HNCO. Additionally, these bands can be grouped into two sets since they behave differently when the matrix temperature increases from 33 K to 35 K (see Figure S2 in Supplementary Materials).

The group of bands denoted as A was characterized by a slight increase in intensity in the temperature range 26–30 K, with a strong intensity increase upon annealing at 33 K and a decrease in intensity upon annealing at 35 K. On the other hand, the intensities of the B-type bands increased over the entire temperature range, but the increase was much stronger between 33 and 35 K. This different behavior of the A and B bands allowed us to assign them to the HNCO dimer and HNCO trimer structures, respectively. 

In Table 4, the selected wavenumber shifts calculated for the five most plausible dimer structures using B3LYPD3 and MP2 methods are compared to the corresponding experimental values. Those obtained at the B2PLYPD3/6-311++G(3df,3pd) level are listed in Table S2 in the Supplementary Materials. The computed intensities of the bands are shown in parentheses in these Tables. Analogous data for HNCO trimers, for the νNH spectral region, are presented in Table S3 in the Supplementary Materials. These computational data were used to support the assignment of the HNCO dimer and trimer species in the following.

The spectral features of group A included several bands located 3519.5, 3465.5, 3462.5, 3452.0 and 3448.5 cm^−1^ (νNH); 2275.0, 2261.5 and 2257.5 cm^−1^ (ν_as_NCO); 788.0, 764.5 and 729.0 cm^−1^ (δHNC) and 599.0 and 560.5 cm^−1^ (δNCO). These band positions and vibrational shifts in relation to the bands of the HNCO monomer, shown in the right panel of Table 4, were in relatively good agreement with the theoretically predicted values for the D2 and D4 dimers. As shown in Figure 1, the D2 and D4 dimers had very similar geometries, both characterized by an elongated structure involving an N-H⋯O hydrogen bond. However, based on obtained experimental and computational results, it is difficult to unequivocally distinguish between these two (HNCO)_2_ forms.

Another set of bands in the spectra of the annealed matrices (group B) included those observed at 3430.0, 3418.0 and 3353.0 cm^−1^ (νNH), 720.0 (δHNC) and 555.5 cm^−1^ (δNCO). Their vibrational shifts relative to the positions of the corresponding HNCO monomer bands were equal to −81.5, −93.5, −158.5, −50.0 and −18.0 cm^−1^, respectively. Formation of trimers, which is expected to increase when annealing the matrix sample at elevated temperatures, most probably takes place by attaching another HNCO monomer to an existing dimer in the matrix based on increasing mobility of molecules in the less restrictive lattice of solid argon. The same process can also result from mobilising isolated HNCO monomers in three-body collisions, but this is less probable compared to the monomer–dimer interaction. 

From the structures shown in Figure 2, while many trimer structures can be formed, the two elongated aggregates T5 and T6 appear to be the most advantageous. Even though these were not the most stable trimer structures computed, the T5 and T6 trimers seem to be the most plausible candidates based on the local or global mobilisation of HNCO molecules in the matrix. Moreover, since the most stable HNCO dimers identified here were linear structures, they will most likely form another hydrogen bond by adding an additional HNCO subunit. Of the two trimer structures, the theoretical shifts in the νNH range calculated for T5 reproduced the experimental results well, and this complex could be the plausible candidate to explain the observed spectral changes upon high temperature annealing cycles.

## 3. Experimental and Computational Details

### 3.1. Matrix Isolation FTIR Studies

Cyanuric acid powder (Acros Organics, Geel, Belgium, 98%) was heated in a quartz vessel under vacuum to T = 450 °C, and the resulting gaseous isocyanic acid was collected in a liquid nitrogen trap. Next, it was passed several times through P_2_O_5_ to remove water traces and was finally stored in a 250 mL glass bulb. The gaseous mixtures were prepared by mixing HNCO with argon (Linde, 5.0, Dublin, Ireland) in a container in a stainless-steel vacuum system. Pressure of the gas mixture and the deposition rate were controlled by piezo transducers (model 902B, MKS Instruments, Andover, MA, USA, accuracy ±1%) installed in the deposition line. Typically, a rate of 2 mbar/min was used. The amount of the deposited mixture varied depending on the concentration of HNCO/Ar from 20 to 120 mbar. Low temperature was obtained using a closed cycle helium cryostat (APD-Cryogenics, Macungie, PA, USA) and measured by a silicon diode sensor coupled with the digital controller (Scientific Instruments, model 9650-1, West Palm Beach, FL, USA). The matrices were deposited onto a CsI window maintained at 15 K. Infrared spectra were collected after cooling the sample to 10 K. Spectra were recorded (averaged over 128 scans) in transmission mode by a Bruker IFS 66 Fourier Transform spectrometer (Bruker Optik, Ettlingen, Germany) equipped with a liquid nitrogen cooled MCT detector with 0.5 cm^−1^ resolution.

### 3.2. Computational Methods

Computational studies for HNCO dimers were performed using the Gaussian16 program package [37]. Structures of the HNCO monomer, dimers and trimers were optimized at the MP2 [38,39,40,41], B3LYP [42,43,44,45], B3LYPD3 [46,47] and B2PLYPD3 [48,49,50] levels of theory using the 6-311++G(3df,3pd) [51,52] basis set. The above methods were chosen because the dispersion interaction is expected to play an important role in the stabilization of the studied complexes. The Cartesian coordinates of optimized HNCO aggregates are provided in Table S4 in Supplementary Materials. Energy optimization of the dimer and trimer structures was conducted with the Boys–Bernardi full counterpoise method by Dannenberg [53,54]. Energies of the optimized structures were further refined by undertaken single-point calculations using the CCSD(T) [55] approach. The interaction energies E_int_ were estimated by subtracting the energies of the isolated monomers with the frozen geometry from the energy of the complexes. The topological analysis of electron density (Atoms in Molecules, AIM) [56] was performed at the MP2/6-311++G(3df,3pd) level using the AIMAll program (Version 19.10.12, Professional) [57] on the HNCO dimers and trimers to recognize the type of interaction present in the studied aggregates. 

Vibrational wavenumbers and intensities were computed at the same levels using the harmonic approximation to confirm that the optimized geometries corresponded to the minima on the potential energy surfaces and to support the analysis of experimental spectra. Spectral shifts upon complexation were obtained as the difference between the aggregate and monomer wavenumbers of the respective modes. To verify the quality of the computational approach for the vibrational data adopted here, a check with higher level theory was performed on the HNCO monomer. The monomer structure was optimized at the CCSD(T)/6-311++G(3df,3pd) level of theory, and the harmonic frequencies obtained thereafter were amended either with B3LYPD3/6-311++G(3df,3pd) or MP2/6-311++G(3df,3pd) anharmonic vibrational corrections. The vibrational frequencies obtained this way were in satisfactory agreement with existing matrix isolation spectrum [12,14] (see Table S5). This gives a plausible confidence in the computed overall vibrational spectra and vibrational shifts of complexed HNCO species considered in the following to help in assigning the vibrational features appearing in the experimental data. We could estimate the role of the anharmonicity on the fundamental vibrational modes primarily for the HNCO monomer species. Based on this, we could approximate that the anharmonicity effects were rather similar for the HNCO subunits in the complexes. This gave us directions for using harmonic calculations and how to assess them with respect to the experimental data obtained.

## 4. Conclusions

Results of computational and experimental FTIR matrix isolation studies of structure and infrared spectra of dimers and trimers of HNCO were presented and discussed. The computational methods used (MP2, B3LYPD3 and B2PLYPD3) revealed five or four stable dimer and nine trimer structures. Based on the results of topological electron density analysis (AIM), it was possible to determine the types of non-covalent interactions in the aggregates under study. The HNCO subunits upon complexation were bound either by N-H⋯N or/and N-H⋯O hydrogen bonds or by van der Waals interactions. Experimentally, HNCO dimers exhibiting the N-H⋯O hydrogen bond were observed and assigned in the spectra of the freshly deposited argon matrices as well as upon low temperature annealing cycles (below 30 K). Annealing at higher temperatures (at 33 and 35 K) led to the formation of new species: HNCO trimers. Both experimental and computational studies indicated that HNCO molecules engage in specific intermolecular interactions, leading to notable changes in infrared spectra. Our results indicate that open, elongated dimers and trimers with N-H⋯O hydrogen bonds are preferred under the conditions of matrix isolation. Therefore, the oxygen atom in the HNCO molecule seems to be a better proton acceptor in solid argon. The observation presented in this work is interesting because such interactions can become essential at low temperatures and appear to be important in molecular aggregation and nucleation processes related to both atmospheric chemistry and astrochemistry. 

## Figures and Tables

**Table 1:** Interatomic distances (Å), angles (degree) and electron density parameters of the intermolecular bond critical points BCP (Atomic units) and ring critical points RCP (Atomic units) of the HNCO dimers computed at the MP2/6-311++G(3df,3pd) level.

Complex	Geometric Parameters ^1^			AIM Parameters		
	Interatomic Distance		Angle	BCP	ρ(r)	∇^2^ρ(r)
	H...Y	X⋯Y	X–H...Y			
D1	2.047	3.055	173.5	H1⋯N6	0.0211	+0.0663
D2	2.039	3.037	169.7	H1⋯O8	0.0170	+0.0698
D3		3.036		C3⋯O8	0.0071	+0.0291
		3.036		C7⋯O4	0.0071	+0.0291
				(4 at.) ^2^	0.0070	+0.0299
D4	2.303	3.062	131.2	H1⋯O8	0.0110	+0.0437
D5		3.196		N2⋯N6	0.0073	+0.0257

^1^ X: N or C; Y: N or O. ^2^ RCP.

**Table 2:** BSSE-corrected interaction energies E_int_ and relative energies ΔE (kJ mol^−1^) of the HNCO dimers calculated at MP2, B3LYP, B3LYPD3, B2PLYPD3 and CCSD(T) levels with 6-311++G(3df,3pd) basis set.

DIMER	MP2		CCSD(T)/ MP2 ^2^		B3LYPD3		CCSD(T)/ B3LYPD3 ^2^		B2PLYPD3 ^1^		CCSD(T)/ B2PLYPD3 ^2^		B3LYP ^1^	
	ΔE	E_int_	ΔE	E_int_	ΔE	E_int_	ΔE	E_int_	ΔE	E_int_	ΔE	E_int_	ΔE	E_int_
D1	0.00	−15.95	0.00	−15.07	0.00	−16.87	0.00	−15.11	0.00	−16.37	0.00	−15.07	0.00	−12.48 (26%)
D2	2.42	−13.36	1.77	−13.65	1.56	−15.11	1.74	−13.82	1.91	−14.28	1.77	−13.69	0.84	−11.43 (24%)
D3	2.47	−13.19	1.06	−13.82	0.74	−15.91	0.90	−14.03	1.85	−14.28	0.98	−13.98	4.05	−8.04 (49%)
D4	3.14	−12.43	2.45	−12.52	2.16	−14.28	2.34	−12.64						
D5	7.92	−7.54	7.66	−6.91	9.17	−7.16	7.63	−6.99	8.88	−6.99	7.63	−6.95	11.03	−0.92 (87%)

^1^ D4 structure is not energetically stable at B2PLYP and B3LYP levels. ^2^ CCSD(T) calculations are single-point calculations at the MP2, B3LYPD3 or B2PLYPD3 optimized geometries, respectively.

**Table 3:** BSSE-corrected interaction energies E_int_ and relative energies ΔE (kJ mol^−1^) of the HNCO trimers calculated at MP2, B3LYPD3, B2PLYPD3 and CCSD(T) levels with 6-311++G(3df,3pd) basis set.

TRIMER	MP2		CCSD(T)/ MP2 ^1^		B3LYPD3		CCSD(T)/ B3LYPD3 ^1^		B2PLYPD3		CCSD(T)/ B2PLYPD3 ^1^	
	ΔE	E_int_	ΔE	E_int_	ΔE	E_int_	ΔE	E_int_	ΔE	E_int_	ΔE	E_int_
T1	0.00	−47.90	0.00	−44.76	0.00	−49.70	0.00	−45.30	0.00	−48.73	0.00	−44.88
T2	3.74	−45.64	3.09	−43.96	0.48	−50.79	3.03	−44.76	2.51	−47.69	2.99	−44.21
T3	4.63	−43.84	3.32	−41.91	2.45	−48.06	3.31	−42.50	3.84	−45.59	3.27	−42.20
T4	6.50	−43.25	5.26	−42.91	2.80	−48.82	5.05	−43.75	4.80	−45.76	5.09	−43.21
T5	15.50	−33.08	13.70	−32.28	14.37	−35.96	13.69	−32.53	14.83	−34.50	13.66	−32.28
T6	18.26	−29.94	15.76	−30.61	16.35	−33.66	15.75	−31.02	17.03	−31.99	15.70	−30.69
T7	17.61	−29.56	15.44	−28.55	16.70	−32.15	15.47	−28.60	17.84	−30.06	15.43	−28.55
T8	19.11	−28.64	16.70	−27.80	18.37	−31.02	16.79	−27.63	19.08	−29.39	16.77	−27.72
T9	26.31	−20.85	23.28	−20.77	25.84	−23.15	23.11	−21.10	26.63	−21.35	23.12	−21.02

^1^ CCSD(T) calculations are single-point calculations at the MP2, B3LYPD3 or B2PLYPD3 optimized geometries, respectively.

**Table 4:** Selected unscaled wavenumber shifts (cm^−1^) of vibrational modes calculated for the HNCO dimers using the B3LYPD3 and MP2 methods with the 6-311++G(3df,3pd) basis set and compared to the experimental results. The calculated infrared intensities (km mol^−1^) of the bands are given in parentheses.

B3LYPD3 ^1^					MP2 ^1^					Mode	Exp. ^2^		Assignment
D1	D2	D3	D4	D5	D1	D2	D3	D4	D5		ν	Δν	
−35	11	−14	0	−2	−38	11	−15	−1	−6	νNH	3519.5	+8.0	D2
(158)	(185)	(324)	(188)	(298)	(174)	(197)	(354)	(207)	(4)				
−165	−78		−48		−143	−62		−50	−6		3465.5 3462.5	−46.0 −49.0 (site)	D2 and D4
(868)	(694)		(253)		(840)	(692)		(259)	(319)		3452.0 3448.5	−59.5 −63.0	
7	11	11	3	11	5	12	10	4	8	ν_as_NCO	2275.0 2261.5	+16.0 +2.5	D2 D4
(544)	(113)	(1365)	(530)	(1319)	(554)	(241)	(1200)	(544)	(1141)				
−8	−2		−1		−7	0		−1			2257.5	−2.0 −1.5	D2 and D4
(1131)	(1658)		(1033)		(912)	(1361)		(858)					
81	19	−8	36	9	84	12	−16	28	14	δHNC	788.0	+18.0	D2
(212)	(247)	(525)	(137)	(396)	(213)	(249)	(528)	(136)	(408)				
30	−41		−26		25	−40		−30			729.0	−41.0	D2
(283)	(266)		(455)		(309)	(286)		(462)					
51	39	−10	33	−5	52	35	−10	25	−3	δNCO	599.0	+25.5	D2
(28)	(67)	(180)	(140)	(103)	(27)	(72)	(220)	(157)	(111)				
25	−6		−4		13	−3		−4			560.5	−13.0	D2
(85)	(113)		(93)		(96)	(134)		(125)					

^1^ The theoretical shifts were calculated relative to the unscaled harmonic frequencies of HNCO monomer at 3681, 2330, 798 and 576 cm^−1^ (B3LYPD3) and 3729, 2337, 785 and 571 cm^−1^ (MP2). ^2^ The experimental shifts were calculated relative to the corresponding monomer band positions at 3511.5, 2259.0, 770.0 and 573.5 cm^−1^, respectively.

## Data Availability

Not applicable.

## Supplementary Materials

The following supporting information can be downloaded at: https://www.mdpi.com/article/10.3390/molecules28031430/s1, Figure S1: The optimized structures of the HNCS dimer [36]; Figure S2: Example plots of integrated intensity versus annealing temperature for bands A and B; Table S1: Interatomic distances (Å), angles (degree) and electron density parameters of the intermolecular bond critical points BCP (Atomic units) and ring critical points RCP (Atomic units) of the HNCO trimers computed at the MP2/6-311++G(3df,3pd) level; Table S2: Selected unscaled wavenumber shifts (cm−1) calculated for the HNCO dimers using the B2PLYPD3 method with the 6-311++G(3df,3pd) basis set. The calculated infrared intensities (km mol−1) of the bands are given in parentheses; Table S3: Selected unscaled wavenumber shifts (cm−1) for the νNH region calculated for the HNCO trimers using the MP2, B3LYP and B2PLYPD3 methods with the 6-311++G(3df,3pd) basis set. The calculated infrared intensities (km mol−1) of the bands are given in parentheses; Table S4: The MP2/6-311++G(3df,3pd) computed Cartesian coordinates (Å) of all optimized HNCO aggregates; Table S5: Harmonic unscaled and anharmonic fundamental vibrational wavenumbers of HNCO, in cm^−1^.

## Sample Availability

Samples of the compounds are not available from the authors.
