# Supplementary material for: Experimental FTIR-MI and Theoretical Studies of Isocyanic Acid Aggregates
Source: Molecules. 2023 Feb 2;28(3):1430. doi: 10.3390/molecules28031430 (PMC9921473; doi:10.3390/molecules28031430)
Supplement: Supplementary file 1 [file molecules-28-01430-s001.zip › molecules-2177936-supplementary.pdf]

Supplementary Materials

Experimental FTIR-MI and theoretical studies of isocyanic acid aggregates

Justyna Krupa, Maria Wierzejewska, Jan Lundell

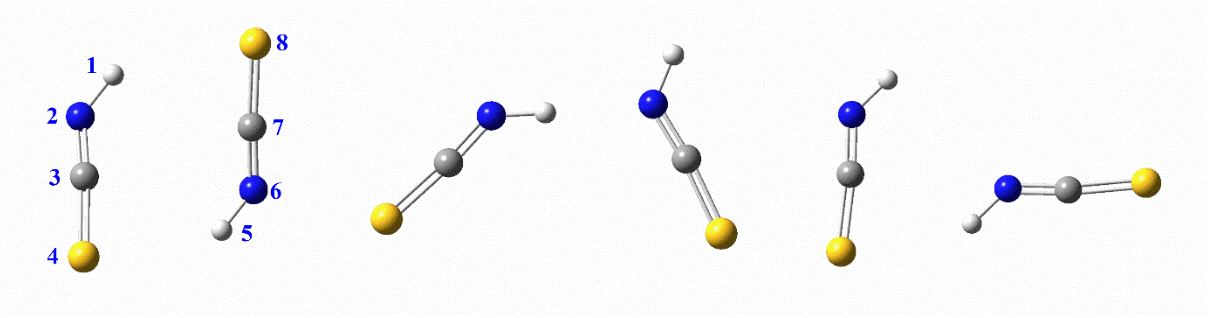

Figure S1. The optimized structures of the HNCS dimer. [36]

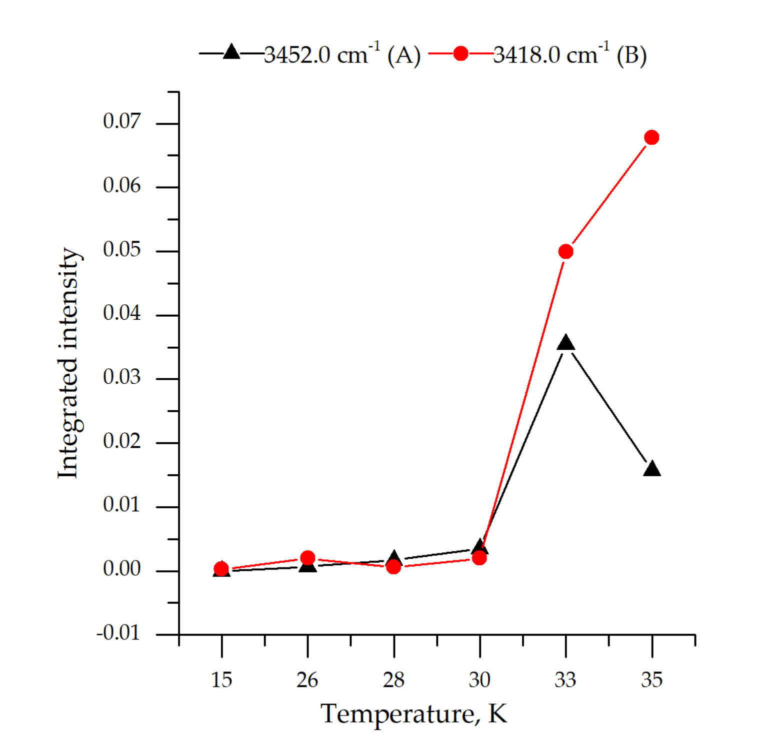

Figure S2. Example plots of integrated intensity versus annealing temperature for bands A and B.

Table S1. Interatomic distances (Å), angles (degree) and electron density parameters of the intermolecular bond critical points BCP (Atomic units) and ring critical points RCP (Atomic units) of the HNCO trimers computed at the MP2/6-311++G(3df,3pd) level.

Complex	Geometric Parameters[1]			AIM Parameters		
	Interatomic distance		Angle	BCP	ϱ(r)	∇^2ϱ(r)
	H···Y	X···Y	X–H···Y			
T1	2.101	2.998	146.1	H1···N6	0.0184	+0.0648
	2.101	2.998	146.1	H5···N10	0.0184	+0.0648
	2.101	2.998	146.1	H9···N2	0.0184	+0.0648
				(6 at.)[2]	0.0049	+0.0241
T2	2.062	3.014	155.6	H1···O8	0.0167	+0.0676
	2.205	3.083	144.6	H5···O12	0.0134	+0.0502
	2.014	3.015	168.0	H9···N2	0.0230	+0.0685
				(10 at.)[2]	0.0014	+0.0064
T3	2.084	3.080	165.6	H1···N6	0.0197	+0.0619
	2.188	3.086	146.8	H5···O12	0.0136	+0.0525
	2.195	3.067	143.3	H9···N2	0.0158	+0.0546
				(8 at.)[2]	0.0025	+0.0113
T4	2.072	3.035	158.7	H1···O8	0.0166	+0.0653
	2.072	3.035	158.7	H5···O12	0.0166	+0.0653
	2.072	3.035	158.7	H9···O4	0.0166	+0.0653
				(12 at.)[2]	0.0003	+0.0014
T5	2.047	3.046	169.7	H1···O8	0.0170	+0.0681
	2.036	3.048	176.5	H5···N10	0.0217	+0.0663
T6	2.052	3.056	173.1	H1···O8	0.0165	+0.0673
	2.039	3.044	174.5	H5···O12	0.0165	+0.0690
T7	2.367	3.110	129.8	H1···O8	0.0093	+0.0373
	2.147	3.154	173.4	H9···N6	0.0167	+0.0559
		3.164		N2···C11	0.0062	+0.0237
				(8 at.)[2]	0.0019	+0.0076
T8	2.079	3.089	175.3	H1···N6	0.0196	+0.0627
		2.997		C7···N10	0.0077	+0.0317
T9		3.076		C3···O8	0.0065	+0.0261
		3.199		N6···N10	0.0073	+0.0258

[1] X: N or C; Y: N or O

[2] RCP

Table S2. Selected unscaled wavenumber shifts (cm^{-1}) calculated for the HNCO dimers using the B2PLYPD3 method with the 6-311++G(3df,3pd) basis set. The calculated infrared intensities ($km\ mol^{-1}$) of the bands are given in parentheses.

B2PLYPD3[1]				Mode
D1	D2	D3	D5	
-36	10	-13	-4	νNH
(159)	(182)	(326)	(297)	
-150	-70			
(833)	(693)			
6	11	10	9	ν_{as}NCO
(536)	(164)	(1252)	(1201)	
-8	-2			
(991)	(1476)			
84	19	-11	12	δHNC
(211)	(252)	(540)	(405)	
29	-40			
(296)	(280)			
49	37	-9	-4	δNCO
(26)	(61)	(173)	(96)	
18	-4			
(82)	(106)			

[1] The theoretical shifts were calculated relative to the unscaled harmonic frequencies of HNCO monomer at 3702, 2319, 803 and 575 cm^{-1}.

Table S3. Selected unscaled wavenumber shifts (cm^{-1}) for the νNH region calculated for the HNCO trimers using the MP2, B3LYP and B2PLYPD3 methods with the 6-311++G(3df,3pd) basis set. The calculated infrared intensities (km mol^{-1}) of the bands are given in parentheses.

B3LYPD3[2]									MP2[2]									B2PLYPD3[2]									Mode	Exp.	
T1	T2	T3	T4	T5	T6	T7	T8	T9	T1	T2	T3	T4	T5	T6	T7	T8	T9	T1	T2	T3	T4	T5	T6	T7	T8	T9		ν	Δν[1]
-204	-65	-124	-92	-43	14	-16	-38	-6	-183	-68	-121	-80	-46	14	-22	-39	-9	-192	-72	-125	-90	-44	13	-18	-37	-7		Group B	
(1158)	(397)	(593)	(789)	(159)	(192)	(181)	(195)	(145)	(1122)	(410)	(735)	(789)	(174)	(201)	(197)	(185)	(163)	(1125)	(406)	(735)	(783)	(160)	(188)	(182)	(175)	(147)		3430.0	-81.5
-204	-151	-148	-92	-105	-85	-46	-49	-16	-183	-138	-136	-80	-87	-62	-52	-56	-19	-192	-147	-141	-90	-96	-74	-50	-51	-17	νNH	3418.0	-93.5
(1158)	(713)	(451)	(789)	(666)	(384)	(247)	(107)	(203)	(1122)	(692)	(344)	(789)	(642)	(440)	(240)	(131)	(258)	(1125)	(706)	(319)	(783)	(649)	(380)	(246)	(118)	(299)		3353.0	-158.5
	-249	-249	-122	-203	-106	-128	-171	-19		-219	-222	-110	-177	-84	-120	-153	-22		-230	-231		-187	-95	-119	-160	-18			
	(892)	(727)	(1)	(1310)	(1379)	(568)	(971)	(104)		(860)	(658)	(0)	(1309)	(1276)	(556)	(953)	(71)		(834)	(671)		(1291)	(1355)	(543)	(942)	(7)			

[1] The experimental shifts were calculated relative to the corresponding monomer band positions at 3511.5 cm^{-1}.

[2] The calculated unscaled harmonic νNH frequency of monomer are: 3681 cm^{-1} (B3LYPD3) 3729 cm^{-1} (MP2) and 3702 cm^{-1} (B2PLYPD3).

Table S4. The MP2/6-311++G(3df,3pd) computed Cartesian coordinates (Å) of all optimized HNCO aggregates.

DIMER

D1
0 1

H	0.49194000	0.66394200	0.21540300
N	1.47471800	0.70488800	0.45171100
C	2.34360600	-0.04366800	0.04838900
O	3.28061200	-0.68044700	-0.25387600
H	-1.79213600	1.77197900	-0.59786800
N	-1.53659600	0.85836300	-0.25866200
C	-2.37009400	-0.01979700	-0.07189200
O	-3.04407800	-0.94428900	0.15039400

D2
0 1

H	4.22527400	-0.81105300	0.25818500
N	3.62944000	-0.18046000	-0.24684500
C	2.49112000	0.12771700	0.03468800
O	1.39618000	0.52655900	0.17189600
H	-0.58828800	-0.13456200	0.12273400
N	-1.47626900	-0.61148100	0.13174400
C	-2.56686700	-0.09320200	-0.01280500
O	-3.67801700	0.25870500	-0.13521000

D3
0 1

H	2.02252500	1.59541700	-0.00002000
N	2.34236400	0.64164700	-0.00008600
C	1.59586700	-0.31794900	-0.00000400
O	1.00514100	-1.33105500	0.00005900
H	-2.02253600	-1.59541500	0.00000600
N	-2.34236900	-0.64164300	0.00004300
C	-1.59586700	0.31794900	0.00000700
O	-1.00513400	1.33105100	-0.00002200

D4
0 1

H	-2.22549900	1.90181400	-0.00007400
N	-2.70422300	1.01839100	0.00006000
C	-2.16600000	-0.06982600	-0.00000500
O	-1.79587900	-1.18330400	-0.00003500
H	0.48485900	-0.86211800	0.00000300
N	1.03571900	-0.01832800	0.00002200
C	2.25407200	0.01942300	-0.00000600
O	3.40734500	0.21609000	-0.00001900

D5
0 1

H	1.35852100	-2.17424500	0.00033200
N	1.02395700	-1.22651300	-0.00040700
C	1.75384200	-0.24852100	-0.00001100
O	2.32284100	0.77277400	0.00016900
H	-1.35852600	2.17424600	0.00047200
N	-1.02395900	1.22651400	-0.00015800
C	-1.75384200	0.24852100	0.00008700
O	-2.32283900	-0.77277500	0.00016700

TRIMER

T1
0 1

H	0.91634700	1.03575700	0.00002900
N	1.68929600	0.37693000	0.00003400
C	2.86822200	0.69566400	-0.00000100
O	4.02517700	0.85581200	-0.00003200
H	-1.35516000	0.27569900	0.00000200
N	-1.17107400	1.27450700	0.00001700
C	-2.03657500	2.13611300	0.00000600
O	-2.75375100	3.05798800	0.00000000
N	-0.51821700	-1.65143000	-0.00000900
C	-0.83164800	-2.83177700	-0.00000600
H	0.43882100	-1.31145600	0.00001000
O	-1.27143200	-3.91380500	-0.00000900

T2
0 1

H	-3.08301800	0.55142600	-0.00513100
N	-2.66914400	1.47144600	-0.00046200
C	-1.49167400	1.74362300	0.00111900
O	-0.38784200	2.15533600	0.00339100
H	1.29944100	0.96919400	0.00338600
N	1.81322000	0.09589700	0.00235800
C	3.03533200	0.03946900	-0.00226700
O	4.18446100	-0.16049100	-0.00646400
N	-0.31292900	-2.04213700	0.01519100
C	-1.48585800	-1.75560800	-0.00001800
H	0.49592800	-1.42814300	0.01016700
O	-2.65626700	-1.61157200	-0.01205500

T3
0 1

H	-0.70176500	-0.38547500	-0.00009700
N	-1.60688500	0.07777800	-0.00004600
C	-2.67692100	-0.51043900	-0.00009900
O	-3.76413300	-0.93885700	-0.00013100
H	1.94398400	-0.03928800	-0.00012400
N	1.33106500	-0.84553200	-0.00012000
C	1.75877200	-1.99295700	-0.00005200
O	2.02131700	-3.12914100	0.00001000
N	-0.19376000	2.80017300	0.00026900
C	0.95073800	2.40509000	0.00014200
H	-0.99992800	2.18750800	0.00019400
O	2.09897100	2.14301800	0.00004000

T4
0 1

H	-1.55563200	2.01498300	0.05978700
N	-0.74066400	2.60170900	0.16451600
C	0.41866300	2.31325900	-0.01175100
O	1.58175800	2.17443800	-0.14273500
H	2.52284400	0.33972700	0.05992200
N	2.62348400	-0.65941600	0.16468000
C	1.79402300	-1.51919600	-0.01164700
O	1.09226100	-2.45705400	-0.14267900
N	-1.88281700	-1.94229100	0.16454400
C	-2.21268500	-0.79406200	-0.01172400
H	-0.96721000	-2.35470300	0.05981800
O	-2.67402200	0.28261300	-0.14270800

T5
0 1

H	-1.95737500	0.67461000	0.56456400
N	-1.16885600	1.30890700	0.51873200
C	-0.08971300	1.15389600	-0.00209700
O	0.99421700	1.14973000	-0.46146600
H	2.66815500	0.03480400	-0.08012700
N	3.37908600	-0.62967600	0.18782100
C	4.57122100	-0.53507000	-0.02954200
O	5.73494700	-0.58374000	-0.16394100
N	-3.53594500	-0.59239000	0.78205700
C	-4.44984500	-0.63581300	-0.03482100
O	-5.23795900	-0.57299100	-0.88976800
H	-3.55039300	-1.15937600	1.61546300

T6
0 1

H	-1.92843700	0.93901000	0.11653900
N	-1.07383400	1.36123800	0.44410200
C	0.05555400	1.20719700	0.04230900
O	1.19758800	1.16639100	-0.23682300
H	2.80249600	-0.09527100	-0.02870400
N	3.51055400	-0.80362400	0.09352600
C	4.71230600	-0.64178400	0.01289400
O	5.88405300	-0.64146800	-0.03244100
N	-5.55901800	-1.31000000	0.11486400
C	-4.66735700	-0.51503200	-0.08325600
H	-6.37331400	-1.18701900	0.68851300
O	-3.73760000	0.13853800	-0.37766900

T7
0 1
H 0.81971400 1.78182700 -0.00435500
N -0.02554500 2.33570200 -0.02909800
C -1.14662700 1.86145900 -0.03019100
O -2.27816700 1.55558100 -0.03539500
H 3.54578400 1.38251400 0.27196400
N 2.73442600 0.81488000 0.09254700
C 2.75083200 -0.40158400 0.00948000
O 2.60710400 -1.55688300 -0.09709900
H 0.26593600 -1.90296300 -0.07727400
N -0.48473000 -1.23271600 -0.01779800
C -1.65974400 -1.57183700 0.02199900
O -2.81234500 -1.75053100 0.06779300

T8
0 1
H 0.28948700 -0.91231800 0.23021500
N -0.29436700 -0.08952200 0.21012300
C 0.21392700 1.01627500 0.08336600
O 0.56054700 2.12474600 -0.03164200
H 3.15707500 1.22725900 -0.16167700
N 3.09965400 0.22443900 -0.08413200
C 4.08226700 -0.50235800 -0.10618900
O 4.91618000 -1.31970900 - 0.11031800
N -3.29931700 -0.57201900 0.73987900
C -4.23611200 -0.48074600 -0.02905500
H -2.32903200 -0.37396000 0.53076600
O -5.22920300 -0.44007300 -0.65168000

T9
0 1
H 0.07646500 -1.91503900 0.00003900
N 0.52748500 -1.01533100 0.00006300
C -0.08436400 0.03663300 0.00005100
O -0.54385800 1.11488600 0.00004300
H 3.29232800 2.09328400 0.00001200
N 2.84020500 1.19540300 -0.00000100
C 3.44790800 0.13637600 0.00000600
O 3.89079600 -0.94517500 0.00001200
N -3.90189900 0.86427400 -0.00006000
C -3.32221000 -0.20501800 -0.00005700
H -3.42202300 1.74877000 -0.00003500
O -2.90385100 -1.30038500 -0.00005900

Table S5. Harmonic unscaled and anharmonic fundamental vibrational wavenumbers of HNCO (cm^{-1}).

Mode	Sym.	Harmonic [1]			Anharmonic [1]				Exp [5]
		B3LYPD3	MP2	CCSD(T)	B3LYPD3	MP2	CCSD(T)/ B3LYPD3 [2]	CCSD(T)/ MP2 [3]	
νNH	A'	3681	3729	3697	3516	3566	3533	3534	3516.8/3505.7
ν_{as}NCO	A'	2330	2337	2304	2291	2301	2265	2268	2259
ν_{s}NCO	A'	1336	1306	1307	1282	1263	1253	1264	n.o.
δHNC	A'	798	785	814	765	758	782	787	769.8
γNCO	A''	639	636	633	662	658	656	655	n.o.
δNCO	A'	576	571	565	584	580	573	574	573.7
MAE [4]		18	17		13	29	10	12	
ǀMAXǀ [4]		29	33		32	54	22	22	

[1] Computed with the 6-311++G(3df,3pd) basis set. [2] Hybrid computations, harmonic wavenumbers computed at the CCSD(T)/6-311++G(3df,3pd) level and anharmonic corrections from B3PLYPD3/6-311++G(3df,3pd). [3] Hybrid computations, harmonic wavenumbers computed at the CCSD(T)/6-311++G(3df,3pd) level and anharmonic corrections from MP2/6-311++G(3df,3pd). [4] Mean (MAE) and maximum (ǀMAXǀ) absolute deviations with respect to the CCSD(T) and experimental values, for the harmonic and anharmonic wavenumbers, respectively. [5] From ref. [14].
